# Comparison of two coracoid process transfer techniques on stress shielding using three-dimensional finite-element model

**DOI:** 10.1186/s13018-022-03264-5

**Published:** 2022-07-30

**Authors:** Seyyid Serif Unsal, Tugrul Yildirim, Murat Kayalar

**Affiliations:** 1 Trabzon Kanuni Research and Education Hospital, Trabzon, Turkey; 2 Present Address: Private Hand Microsurgery Orthopedics Traumatology (EMOT) Hospital, Kahramanlar District, Street 1418, No:14, Konak, Izmir, Turkey

**Keywords:** Latarjet procedure, Congruent-arc Latarjet procedure, CT-based finite-element model, Stress shielding, Osteolysis

## Abstract

**Background:**

We created patient-based 3D finite-element (FE) models that simulate the congruent-arc Latarjet (CAL) and traditional Latarjet (TL) procedures and then compared their stress distribution patterns with different arm positions and glenoid defects.

**Methods:**

The computed tomography data of 10 adult patients (9 men and 1 woman, ages: 18–50 years) were used to develop the 3D FE glenohumeral joint models. Twenty-five and 35% bony defects were created on the anterior glenoid rim, and the coracoid process was transferred flush with the glenoid by the traditional and congruent-arc techniques using two half-threaded screws. A load was applied to the greater tuberosity toward the center of the glenoid, and a tensile force (20 N) was applied to the coracoid tip along the direction of the conjoint tendon. The distribution patterns of the von Mises stress in the traditional and congruent-arc Latarjet techniques were compared.

**Results:**

The mean von Mises on the graft was significantly greater for the TL technique than for the CAL. While the von Mises stress was greater in the distal medial part of the graft in the TL models, a higher stress concentration was observed in the distal lateral edge of the coracoid graft in the CAL models. The proximal medial part of the graft exhibited significantly lower von Mises stress than the distal medial part when compared according to technique, defect size, and arm position. Increasing the glenoid defect from 25 to 35% resulted in a significant increase in stress on the lateral side of the graft in both models.

**Conclusion:**

The stress distribution patterns and stress magnitude of the coracoid grafts differed according to the procedure. Due to placing less stress on the proximal–medial part of the graft, the CAL technique may lead to insufficient stimulation for bone formation at the graft–glenoid interface, resulting in a higher incidence of graft osteolysis.

*Clinical relevance* The CAL technique may lead to a higher incidence of graft osteolysis.

**Level of evidence:**

Basic Science Study; Computer Modeling.

## Introduction

The Latarjet procedure, also known as the coracoid process transfer, is one of the most performed and reliable procedures for treating recurrent anterior shoulder instability in patients with a significant bone defect of the glenoid surface [1–4]. In the traditional Latarjet (TL) procedure, the inferior surface of the coracoid process (CP) is transferred with the conjoint tendon to the anterior glenoid rim. A “sling effect” is created by the conjoint and subscapularis tendons, and the bony prominence of the transferred coracoid and the ligament effect of the coracoacromial ligaments provide anterior stability [5, 6]. However, the extent to which these effects contribute to stability, at different degrees of abduction and external rotation, is still being debated [7, 8].

Multiple technique modifications to the Latarjet procedure have been reported. For example, Burkhart et al. proposed the “congruent-arc Latarjet’’ (CAL) technique, in which the coracoid is rotated 90° along its longitudinal axis, allowing the medial surface of the coracoid to be fixed to the anteroinferior of the glenoid and making the inferior surface compatible with the articular surface of the glenoid [3, 9]. Potential advantages of the CAL technique are that it provides a wider surface on which to restore the glenoid defect, it has a slope closer to that of the natural glenoid, and it reduces contact stress around the glenohumeral joint [10–12]. However, multiple biomechanical studies have addressed poor fixation stability as a potential weakness of the CAL technique [13, 14].

Graft osteolysis is a complication of the Latarjet procedure [15, 16]. According to Wolff’s law, if loading on a bone increases, the bone will remodel itself over time, and if the loading on a bone decreases, the bone will become weaker. The screws inserted during the Latarjet procedure contribute to the stress shielding in the proximal part of the coracoid graft, which eventually leads to osteolysis in the proximal part [7, 17–19].

A computed tomography (CT)-based 3D finite-element (FE) method has been widely used to assess the stress distribution within the bone to describe the risk of osteolysis [20–23]. By precisely reflecting the bone’s architecture, the 3D FE model can visualize the stress distribution inside the bone. Although several studies have investigated the stress distribution in the coracoid graft with a 3D FE model, a comparison of the stress distribution between the CAL and standard TL techniques has not been conducted [18, 19].

In this study, we created patient-based 3D FE models that simulate the CAL and TL procedures, and then compared their stress distribution patterns in different arm positions and glenoid defects. We hypothesized that there would be significant differences in the stress distribution patterns of the two techniques.

## Materials and methods

### Development of the FE defect model

For the creation of the study’s humerus and scapula bone model, the CT data of 10 adult patients (9 males and 1 female, age: 18–50 years) without shoulder trauma were used. The CT data were reconstructed with a slice thickness of 0.1 mm and were transferred to the 3D Slicer software in the DICOM (.dcm) format. The CT data in DICOM format were separated according to appropriate Hounsfield values using 3D Slicer software and converted into a 3D model by segmentation. The model was exported in an.stl format.

Arrangement of the 3D mesh structure and its transformation into a mathematically appropriate solid mesh structure creation of 3D FE analysis models and FE stress analysis were performed on HP workstations equipped with an INTEL Xeon E-2286 processor (2.40 GHz and 64 GB ECC memory). The 3D.stl model of the bone structure created from the CT data was obtained using 3D Slicer software. Reverse engineering and 3D CAD activities were carried out with Altair Evolve software; the Nastran-based Altair Optistruct (Altair, Troy, MI, USA) implicit solver was used to solve the FE models. The mathematical mesh models and the force transfer between the models were created in Altair HyperMesh. The prepared models were placed in the correct 3D space coordinates via the Altair Evolve software, and the modeling process was completed.

**Fig. 1:**
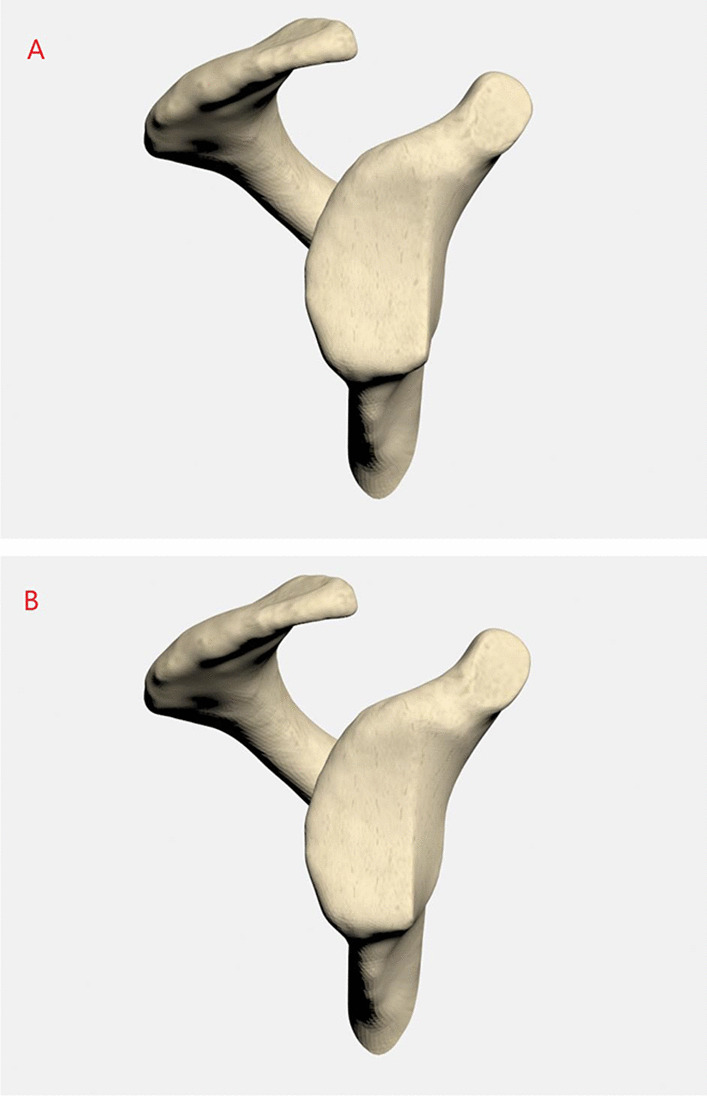
**A.** 25% defect model. **B.** 35% defect model

The maximum glenoid defect size that could be restored by a TL graft ranged between 19.2% and 38.8% [21]. A maximum defect size of 35% does not exceed the coverage capacity of the TL, while a minimum defect size of 25% necessitates reconstruction of the glenoid bone stock [24, 25] (Fig. 1).

The defect was set parallel to the longitudinal axis of the glenoid according to previous studies [19, 25]. The distal part of the coracoid process (length: 2.5 cm) was resected for simulating the coracoid osteotomy. Cartilage tissue was modeled with reference to the outer surface of the cortical bone, and the trabecular bone was modeled with reference to the inner surface of the cortical bone. On the basis of previous studies, the articular cartilage thickness was determined to be 2.0 mm in both the glenoid and the humeral head [19, 26].

### Simulation of the coracoid transfer

The inferior part of the coracoid was transferred to the anterior glenoid defect to simulate the TL method. To simulate the congruent-arc method, the graft was rotated 90° around the y-axis. Two half-threaded titanium screws (titanium alloy: Ti-6AI-4 V, diameter: 3.5 mm, length: 35 mm) were created similar to commercial models (Depuy Synthes, Raynham, MA, USA) and were used to fix the coracoid process. Care was taken to ensure that the coracoid process was placed flush with the glenoid cartilage. Soft tissues were not modeled in this study.

### Arm position

**Fig. 2:**
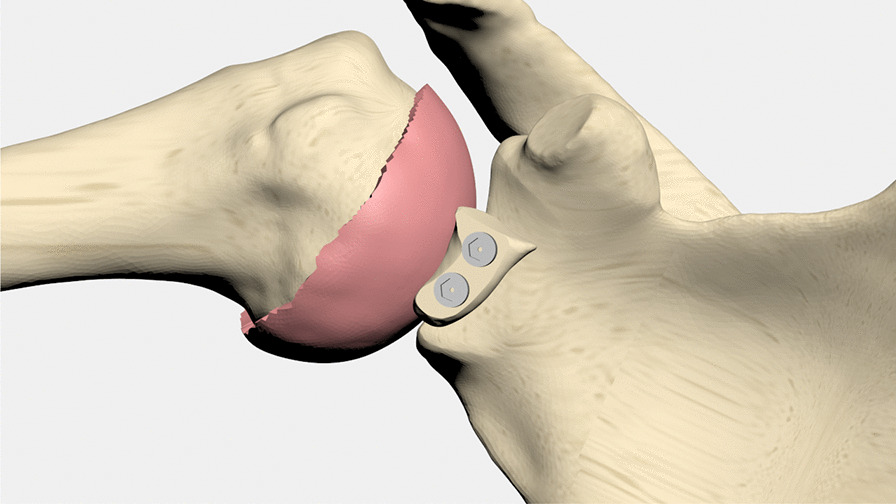
90^0^ abduction position. The center of the humeral head was determined to be the center of rotation. The abduction angles of the scapula and humerus were 30° and 60°, respectively

The initial FE models were created at the hanging arm position in a neutral position. To clarify the stress distribution pattern, a 90° shoulder abduction position was simulated according to previous studies [19, 27] (Fig. 2). The center of the humeral head was determined to be the center of rotation. The abduction angles of the scapula and humerus were 30° and 60°, respectively.

### Contact conditions

In all models, the screw–bone (cortical and trabecular) interface was modeled with friction contact using a coefficient of μ = 0.3; therefore, all models were run nonlinearly, with the exception of the freeze-type contact that was defined in all contact areas (cortical–trabecular, cortical–cortical, and cortical–cartilage) [28]. This approach is based on the assumption that there is no slip at the pre-stressing interfaces and that the parts move with full correlation during their movement.

### Material properties

The linear material properties of the materials were used in the analysis [18, 19]. The elastic modulus and Poisson ratio values of the materials are presented in Table 1.

**Table 1:** Material properties

Material	Elastic modulus (MPa)	Poisson ratio
Humerus cortical bone	13,400	0.3
Humerus trabecular bone	2000	0.3
Scapula cortical bone	10,000	0.3
Scapula trabecular bone	1000	0.4
Titanium screw	113,800	0.3
Biceps tendon	35	0.49

### Boundary conditions

A force of 50 N was applied from the humeral bone head to the glenoid region of the scapula [13, 18, 25]. To simulate the pulling force of the relevant tendon, a force of 20 N was applied from the tip of the coracoid graft to the humerus [18, 19]. The models were fixed by limiting all degrees of freedom from the nodal points located in the distal region of the humeral cortical and trabecular bone and in the medial region of the scapula cortical and trabecular bone.

### FE analysis and data interpretation

All models were subjected to elastic analysis by visualizing the distribution pattern of von Mises stress (VMS). Each coracoid graft was divided into four parts (proximal–medial, proximal–lateral, distal–medial, and distal–lateral) to specifically identify the mean VMS of these areas. The stress distribution over the TL and CAL grafts was analyzed for different defect sizes to compare the extent of the stress shielding associated with each procedure.

### Statistical analysis

The data analysis was performed with SPSS 26.0, and the data were studied with a 95% confidence level. Because of the small sample size, nonparametric methods were used in the study, as well as Mann–Whitney and Kruskal–Wallis tests.

## Results

**Fig. 3:**
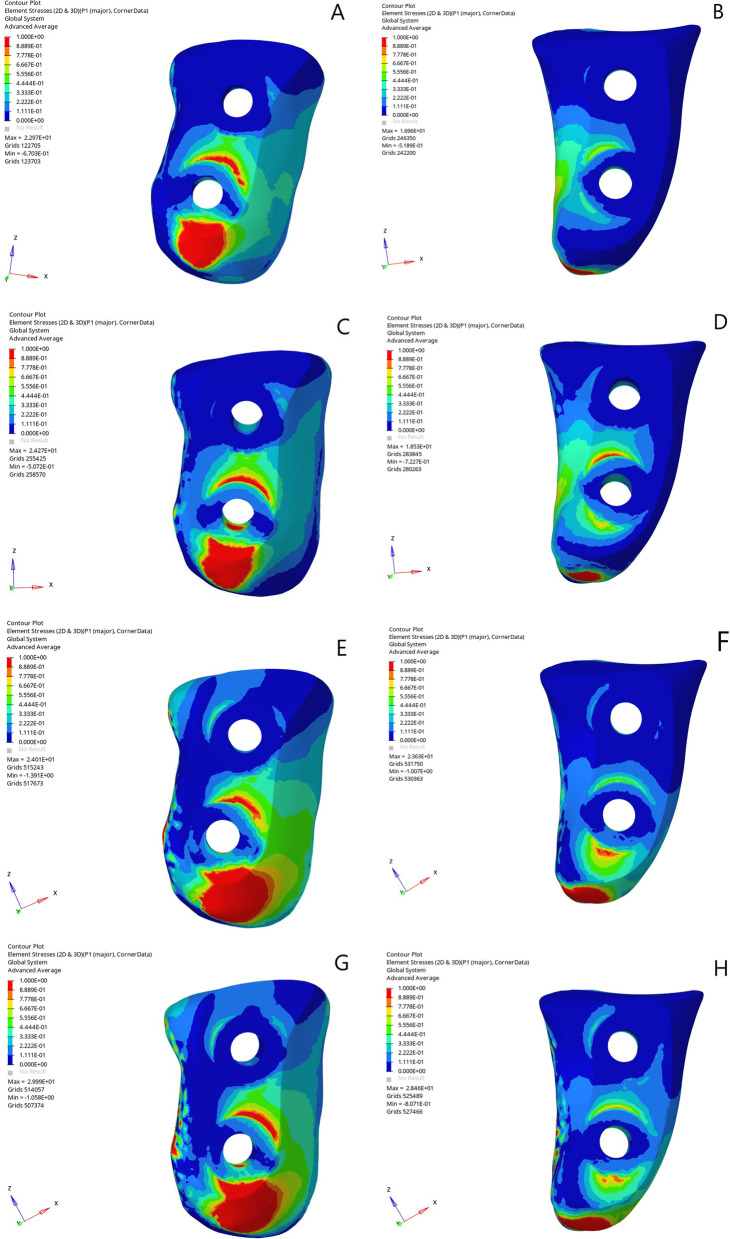
**A.** TL, 25% glenoid defect, neutral arm position. **B.** CAL, 35% glenoid defect, neutral arm position. **C.**TL, 35% glenoid defect, neutral arm position. **D.** CAL, 35% glenoid defect, neutral arm position. **E**. TL, 25% glenoid defect, 90^0^ abduction position. **F.** CAL, 25% glenoid defect, 90^0^ abduction position. **G.**TL, 35% glenoid defect, 90^0^ abduction position. **H.** CAL, 35% glenoid defect, 90^0^ abduction position

The mean VMS values for all scenarios are summarized in Fig. 3.

**Fig. 4:**
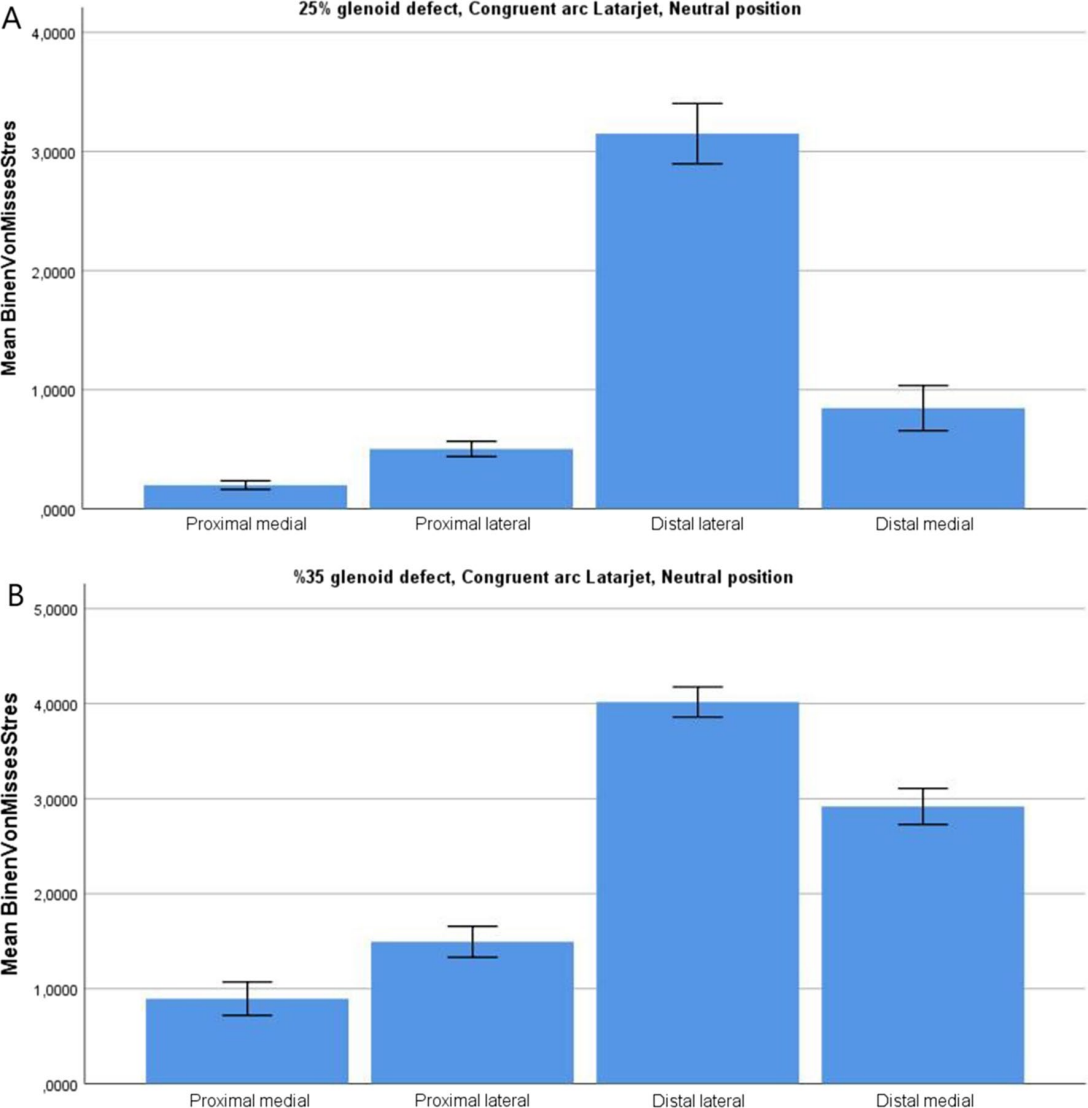
**A.** Distal lateral part of the graft exhibited the most stress in 25% glenoid defect CAL model. **B.** When the defect size was increased to 35%, distal medial part of the graft exhibited more stress

The mean VMS on the graft was significantly greater in the TL technique than in the CAL technique in the 25 and 35% defect models (*p* > 0.05). The stress distribution patterns of the coracoids differed according to the procedure: while the VM stress was greater in the distal medial part of the graft in the TL models, a higher stress concentration was observed in the distal lateral edge of the coracoid graft in the CAL models (*p* > 0.05) (Fig. 4). In all models, the proximal part of the grafts exhibited significantly less VMS (*p* > 0.05).

Increasing the glenoid defect from 25 to 35% resulted in a significant increase in stress on the lateral side of the graft in both models (*p* > 0.05). However, there was no significant increase or decrease in the VMS in the remaining parts of the graft for either technique. In the 90° abduction models, significantly greater VMS was observed in both the CAL and TL grafts than in the neutral position models (*p* > 0.05).

## Discussion

To our knowledge, this is the first study comparing stress distribution patterns between the TL and the CAL procedures. Since the Latarjet procedure is a non-anatomical augmentation of the glenoid surface, it can alter the intra-articular stress distribution dramatically. Previous biomechanical and FE model studies have shown that graft osteolysis is expected in the proximal part of the graft in the LT technique [18, 19]. However, no studies have been performed to describe the stress distribution and its magnitude in the CAL procedure. This study clearly demonstrated, as we expected, that the stress distribution patterns and stress magnitude of the coracoid grafts differ according to the procedure. These differences may be attributed to the increased surface contact with the humeral head in the CAL model, which may have resulted in less mean stress on the graft and relatively greater stress concentration on the lateral edge. Alternatively, with the TL model, higher stress was observed in the medial part of the graft where bony consolidation was expected. The lack of mechanical stimuli in certain areas of the coracoid graft can contribute to osteolysis in these areas. [7, 29]

That the medial stress is less in the CAL technique than in the TL may cause insufficient stimulation for bone formation at the graft–glenoid interface, which could eventually lead to a greater incidence of graft osteolysis or nonunion. Mengers et al. analyzed 26 studies comparing TL and CAL techniques, identifying that the fibrous or nonunion incidence was greater for the CAL technique [30]. Graft osteolysis may occur if a larger than necessary graft is used because the graft does not experience adequate forces from the humeral head and subsequently resorbs in accordance with Wolff’s law [17]. Some authors have proposed that when a smaller defect is filled using the CAL technique, the width of the graft size should be reduced to prevent stress shielding [14]. We observed a significant increase in stress on the CAL graft when the glenoid defect was increased from 25 to 35%. Previous studies have reported that up to 53% of the glenoid may be restored using the CAL technique. Considering our findings and previous studies, the CAL technique should not be used for small defects.

Less stress was observed at the proximal half of the coracoid bone graft in both the TL and CAL techniques for both glenoid defects and arm positions. A high stress concentration was identified at the distal part of the coracoid graft caused by the tensile force created by the conjoint tendon. Similar to the findings of Sano et al., the proximal–medial part represented the lowest equivalent stress of the four parts of the coracoid graft in both models [18, 19]. The insertion of two screws may have shielded the proximal half of the coracoid from the tensile force of the conjoint tendon. These findings indicate that the proximal–medial part of the graft may have the highest risk of osteolysis, regardless of the technique.

Latarjet is one of the most widely used surgical procedures for treating significant bone defects of the glenoid surface and failed Bankart repairs [2–4]. Although the TL procedure is effective for the management of recurrent anterior shoulder instability, it is not without complications. Coracoid graft osteolysis and fibrous nonunions are considered the main causes of recurrent dislocation, pain, and stiffness in patients after the Latarjet procedure [7, 31]. Hurley et al. evaluated 13 studies with a minimum follow-up of 10 years in patients who underwent the TL procedure and found 8.5% dislocation recurrence and 3.7% revision rates [16]. To replace the articular shape of the glenoid, Burkhart et al. proposed the “congruent-arc’’ technique, in which the graft is rotated 90° along its longitudinal axis, thus allowing the medial surface of the coracoid to fix to the anteroinferior of the glenoid and making the inferior surface compatible with the articular surface of the glenoid [3]. Because the radius of the curvature of the inferior face of the coracoid graft is similar to that of the native glenoid, it is possible to reconstruct larger glenoid defects, providing a more significant bone-blocking effect and decreasing the contact pressure across the glenohumeral joint [10, 13, 32, 33]. In a recent meta-analysis comparing the TL and CAL modifications, patients undergoing CAL were less likely to have recurrent subluxation, postoperative complications, and reoperations and were more likely to return to sports and activities. [30]

The small contact area between the graft and glenoid makes the CAL technique significantly less resistant to load to failure compared with the TL tchnique [13, 14]. In addition, there is a shorter bone distance around the screw with the CAL technique, which makes it very difficult to perform in patients with small coracoids [34]. Moreover, a higher incidence of broken, loose, or improperly placed screws have been reported with the CAL technique, although no difference was observed in graft positioning [30]. Male patients were significantly more likely to undergo augmentation using the CAL technique. When deciding on which technique to use, the patient’s coracoid width and size of the glenoid defect should be considered.

This study has the following limitations. First, we did not perform the analysis on a 3D dynamic motion model of the shoulder joint. Future studies, including dynamic analyses, are necessary to describe the true biomechanical environment created following the TL and CAL techniques. Second, there were numerous assumptions with respect to the material properties, as well as the boundary and contact conditions, each of which may have affected the results. Third, our results were not subjected to further biomechanical validation because of the technical difficulties associated with measuring the actual stress distribution during cadaveric testing. However, because our results are consistent with those of previous studies, we believe our simulation is an effective and accurate recreation of real-world conditions.

## Conclusion

The TL and CAL techniques exhibited different stress distribution patterns and stress magnitudes on coracoid grafts. Although the CAL modification provides a more significant bone-blocking effect and decreases the contact pressure across the glenohumeral joint, less stress on the medial part of the graft may lead to insufficient stimulation for bone formation at the graft–glenoid interface, which could eventually lead to a higher incidence of graft osteolysis. Surgeons should be aware of this risk when considering the CAL technique, especially for small glenoid defects not exceeding 35% of glenoid width.

## Data Availability

The data underlying the current study will be shared by the corresponding author on reasonable request.

## Abbreviations

FE: Finite element
TL: Traditional Latarjet
CAL: Congruent-arc Latarjet
VMS: Von Mises stress
CT: Computed tomography
CP: Coracoid process
